# Enhanced nasopharyngeal infection and shedding associated with an epidemic lineage of *emm*3 group A *Streptococcus*

**DOI:** 10.1080/21505594.2017.1325070

**Published:** 2017-05-01

**Authors:** Baharak Afshar, Claire E. Turner, Theresa L. Lamagni, Ken C. Smith, Ali Al-Shahib, Anthony Underwood, Matthew T. G. Holden, Androulla Efstratiou, Shiranee Sriskandan

**Affiliations:** a Department of Medicine, Imperial College London, London, U.K; b National Infection Service, Public Health England, London, U.K; c Pathology and Pathogen Biology, Royal Veterinary College, University of London, Hertfordshire, U.K; d Pathogen Genomics, The Wellcome Trust Sanger Institute, Wellcome Trust Genome Campus, Hinxton, Cambridge, U.K; e School of Medicine, University of St Andrews, St. Andrews, U.K

**Keywords:** DNases, epidemic upsurge, group A *Streptococcus*, genotype *emm*3, invasive group A streptococcal disease, nasopharyngeal infection, prophage, serotype M3, superantigens, *Streptococcus pyogenes*

## Abstract

**Background:** A group A *Streptococcus* (GAS) lineage of genotype *emm*3, sequence type 15 (ST15) was associated with a 6 month upsurge in invasive GAS disease in the UK. The epidemic lineage (Lineage C) had lost 2 typical *emm*3 prophages, Φ315.1 and Φ315.2 associated with the superantigen *ssa*, but gained a different prophage (ΦUK-M3.1) associated with a different superantigen, *speC* and a DNAse *spd1*. **Methods and Results:** The presence of *speC* and *spd1* in Lineage C ST15 strains enhanced both *in vitro* mitogenic and DNase activities over non-Lineage C ST15 strains. Invasive disease models in *Galleria mellonella* and SPEC-sensitive transgenic mice, revealed no difference in overall invasiveness of Lineage C ST15 strains compared with non-Lineage C ST15 strains, consistent with clinical and epidemiological analysis. Lineage C strains did however markedly prolong murine nasal infection with enhanced nasal and airborne shedding compared with non-Lineage C strains. Deletion of *speC* or *spd1* in 2 Lineage C strains identified a possible role for *spd1* in airborne shedding from the murine nasopharynx. **Conclusions:** Nasopharyngeal infection and shedding of Lineage C strains was enhanced compared with non-Lineage C strains and this was, in part, mediated by the gain of the DNase *spd1* through prophage acquisition.

## Background

Group A *Streptococcus* (GAS) has the ability to cause a diverse range of clinical manifestations. Periodic upsurges in invasive GAS (iGAS) disease can occur at a national level and are often associated with expansion of a single genotype within the population.^1-3^ Although recombination-related remodelling in the core genome can lead to sustained expansion of GAS lineages,^1^ the biologic basis for short-lived upsurges is unclear.

**Figure 1.**
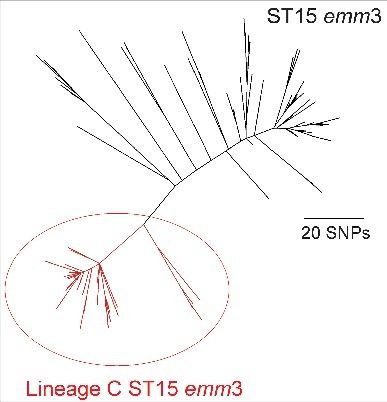
Phylogenetic representation of the ST15 *emm*3 population. Within the ST15 population (indicated in black) was the identified epidemic Lineage C ST15 (indicated in red), characterized by the unique prophage profile; Φ315.1 and Φ315.2 are absent in all 78 Lineage C strains and 65/78 carried ΦUK-M3.1 with *speC* and *spd1*. The short reads for 172 ST15 isolates^6^ were mapped to the ST15 reference strain MGAS315 and concatenated SNPs from the core genome (excluding all prophage regions) were used to generate the maximum likelihood phylogenetic tree with RAxML^7^.

In the United Kingdom an upsurge in iGAS was recorded in the 6 months between November 2008 and April 2009, prompting a public health alert.^4^ Analyses of the collected epidemiological data did not identify increases in any particular risk group or iGAS manifestation.^5^ A large proportion of the upsurge could however be attributed to a marked increase in the proportion of *emm*3 iGAS infections and, more specifically to a new lineage of sequence type (ST) 15 within the *emm*3 population with an altered genomic prophage profile, identified by whole genome sequencing^6^ (Fig. 1). This lineage was first identified in the UK *emm*3 population in 2006 and rapidly increased in prevalence during the upsurge period, before declining again to be undetectable by 2013.^6^ Unlike typical *emm*3 ST15 GAS strains, ST15 strains belonging to this lineage (Lineage C) had lost 2 prophages; Φ315.1 with no known associated virulence factors, and Φ315.2 that is associated with the superantigen *ssa*. Lineage C strains had also gained a different prophage (ΦUK-M3.1) that carried the superantigen *speC* and a DNase, *spd1*, rarely found in *emm*3 before the upsurge. Here, we report the phenotypic impact of this genetic change on experimental and clinical disease, to better understand the mechanisms that allow a sudden but transient emergence of new lineages and periodic increases in invasive infection. Interestingly, we identified that the genetic changes that occurred in Lineage C ST15 strains compared with other ST15 Lineages, did not impact on experimental invasiveness but notably enhanced nasopharyngeal infection and shedding.

## Material and methods

### Ethics statement

Normal donor cells were acquired from an approved sub-collection of the Imperial College Tissue Bank. In vivo experiments were performed in accordance with the Animals (Scientific Procedures) Act 1986, and were approved by the Imperial College Ethical Review Process panel and the UK Home Office.

### Bacterial culture

GAS isolates were grown in Todd-Hewitt broth (THB) (Oxoid, UK) or Columbia horse blood agar. For *in vitro* mitogenicity testing, GAS were cultured in RMPI (Life Technologies) +10% foetal calf serum, and cell-free supernatants were obtained by 0.25 μM filtering before co-incubation with cultured human MNC or murine spleen cells.

### Selection of GAS strains for experimental phenotyping

Lineage C was identified following a whole genome sequencing (WGS) study of 442 *emm*3 invasive and non-invasive strains from the UK and Ireland, isolated 2001–2013.^6^ Isolate selection was described by Al-Shahib et al.,^6^ and encompassed the upsurge period, a previous period of enhanced surveillance and an even distribution across 2001–2013. Phenotypic comparisons were limited to ST15 *emm*3 strains only, to focus on the novel Lineage C strains. Of the 442 sequenced, 78 were Lineage C ST15 *emm*3 and 98 were non-Lineage C ST15 *emm*3 (Fig. 1). All 78 Lineage C strains had lost Φ315.1 and Φ315.2 and 65/78 had gained ΦUK-M3.1, however no ΦUK-M3.1 negative Lineage C strains were used in any study. For *in vitro* study, we selected ST15 isolates carrying only the superantigens *speA, speG* and *speK* in addition to *ssa* or *speC*, from all the ST15 isolates that had been WGS at that time. This included all ST15 from a collection of 200 *emm*3 strains of different STs isolated in 2008–2009 and the previous period of enhanced surveillance (2003–2004) (as described by Al-Shahib et al.) plus a further 95 isolates across years 2001–2007 and 2010–2011. The result was 51 Lineage C strains and 41 non-Lineage strains.

For pilot *Galleria mellonella* studies, 60 isolates were originally selected before WGS, and included 42 from 2008–2009 and 18 from 2004 (to act as a pre-upsurge comparison). Following WGS we able to select only isolates that were ST15 and excluded any carrying mutations within the regulatory system *covRS*, with the exception of Lineage C strains which all carry a non-synonymous SNP changing glutamic acid to glycine at amino acid 252/500 (E252G). No lineage-specific difference in the expression of the CovRS positively regulated protease SpeB was observed between Lineage C and non-Lineage C ST15 strains, as determined by Western blotting (Fig. S1), suggesting this amino acid substitution does not affect CovS function.

Two Lineage C ST15 strains (M3-C1, M3-C2) and 2 non-Lineage C ST15 strains (M3–1, M3–2) were selected for murine *in vivo* work as representatives of each type (Fig. S2), blind to any phenotypic data, and paired based on fewest number of SNPs outside of Lineage-associated SNPs. These 4 strains do not carry mutations within known virulence related genes and regulators (CovRS, Rgg1–4, FasABCX, RivR) with the exception of the CovS E252G found in M3-C1 and M3-C2, and all other Lineage C strains.

### Western blot analysis

Overnight THB culture supernatant from Lineage C and non-Lineage C ST15 GAS strains was 0.25 µM filtered before concentrating 16-fold using TCA precipitation. Proteins were separated by 10% Bis-Tris LDS-PAGE (Life Technologies) and immunoblotted using rabbit anti-SPEC antibody (Toxin Technology), rabbit anti-SPEB antibody (Toxin Technology) or rabbit anti-SPEA C-terminal antibody. Quantification of SPEC was performed using densitometry (ImageJ) and protein concentration calculated by comparison with standard concentrations of recombinant SPEC (Toxin Technology). No lineage-associated differences between Lineage C and non-Lineage C strains were observed for SPEA and SPEB expression (Fig. S1).

### *Pilot Galleria mellonella* (waxmoth larvae) virulence assay

To screen for overall virulence differences, survival assays were conducted with groups of 10 *Galleria mellonella* larvae (Livefoods Direct Ltd, Sheffield, UK) infected with each of 7 non-Lineage C ST15 *emm*3 strains or each of 5 Lineage C-associated ST15 *emm*3 strains. Larvae were injected via the bottom left proleg (10^5^–10^6^ CFU/larvae) and incubated overnight at 37°C. Larvae were scored for survival at 14h, 24h, then daily up to 120h. The entire experiment was repeated on a second batch of larvae.

### DNase activity

Overnight THB culture supernatant from ST15 GAS isolates was 0.25µM filtered and inoculated into wells cut into methyl green agar (Oxoid). Plates were incubated overnight at 37°C and the diameter of DNA hydrolysis was measured. Activity was calculated by comparison with known dilutions of DNase I (Life Technologies). All samples were tested in duplicate.

### Mitogenicity assays

Proliferation of human MNC or spleen cells from HLA-DR4 (Taconic) or HLA-DQ8 transgenic humanized mice was measured based on tritiated thymidine uptake after 72h co-incubation with 1:100 diluted filtered bacterial culture supernatant, recombinant superantigen, 10% mouse sera or infected tissue as described previously.^8^

### Recombinant expression and purification of SPEC and SSA

Recombinant SPEC and SSA were expressed using pGEX-2T constructs kindly provided by Thomas Proft (University of Auckland)^9,10^ and purified from IPTG-induced BL21 *E. coli* via GSH-agarose columns. The GST fusion protein was removed from rSPEC and rSSA by 5 µg/ml 3c protease or trypsin.^9^

### Murine intramuscular infection

HLA-DQ8 female mice were infected intramuscularly (thigh) with 10^6^ – 10^7^ (50µl) CFU of GAS and after 24h of infection, mice were killed. Blood was taken by cardiac puncture. Spleen, liver, local draining lymph node and infected thigh muscle were excised, individually weighed and homogenized in sterile PBS before plating to quantify viable bacterial counts. Serum was collected by centrifugation and stored together with infected thigh tissue homogenate at −20°C for mitogenic activity and cytokine analysis.

### Murine intranasal infection and shedding

An inoculum of 10^6^ – 10^7^ CFU in 10 µl was administered to female HLA-DQ8 mice via the intranasal route under brief isoflurane anesthesia. Nasal shedding of GAS was monitored daily for 7 d using a nose-pressing technique.^11^ To detect airborne shedding of GAS within cages of infected mice, CBA plates were placed in the upper rack of the individually HEPA-filtered cages (4 plates per cage) and exposed for 4h as described previously.^11^ Plates were then incubated overnight at 37°C, 5% CO_2_ and the number of GAS colonies (identified by β-hemolysis and Lancefield grouping) recorded. This procedure was repeated every 24h for 4 d. For histopathology, the entire nasopharynx was removed, fixed in formalin and processed as described previously.^11^ Inflammation was scored by a blinded histopathologist (KS).

### Cytokine and chemokine measurement

Murine cytokines/chemokines were measured in infected serum, nasal tissue and thigh tissue homogenate using a mouse cytokine Luminex® 20-plex panel (Life Technologies) and analyzed on a Bio-Rad Bio-Plex 200 system.

### Construction of allelic exchange mutants

To construct *speC* exchange mutants, a 670bp region directly upstream of the *speC* gene was amplified (forward primer: 5′ – *GGGGTACCAACTATTTTGATGATGTTAATC*, reverse primer: 5′ –*CGGAATTCCCATATCCAACTGCAAGATAG*) incorporating KpnI and EcoRI restriction sites at either end and cloned into the suicide vector pUCMUT upstream of the *aphA3* kanamycin resistance cassette. A 643bp region directly downstream of the *speC* gene was amplified (forward primer: 5′ – *AAAACTGCAGGAAGTCCATCGCATGCCGAC*, reverse primer: 5′ – *ACGCGTCGACAGTCATTTCGATATTTATCTTG*) incorporating PstI and SalI restriction sites at either end and cloned into pUCMUT downstream of the *aphA3* kanamycin resistance cassette. Similarly, to construct *spd1* exchange mutants, a 685 bp region directly upstream of *spd1* was amplified (forward primer: 5′ – *GGGGTACCTGCGTCAACAGTTATTGTCG*, reverse primer: 5′ – *CGGAATTCCACATTAGCCTCGTTCACGC*) and cloned in pUCMUT upstream of the *aphA3* kanamycin resistance cassette. A 711 bp region directly downstream of *spd1* was amplified (forward primer: 5′ – *AAAACTGCAGCCGCTTACATAAGGAGAAG*, reverse primer: 5′ – *ACGCGTCGACCTCCAAATATCAACCTTGAC*) and cloned into pUCMUT downstream of the *aphA3* kanamycin resistance cassette creating pUCMUT_spd1_. The resultant vectors pUCMUT_speC_ and pUCMUT_spd1_ were then introduced into 2 different Lineage C ST15 *emm*3 strains (M3-C1 and M3-C2) by electroporation, selected by 400 µg/ml kanamycin and allelic exchange confirmed by PCR and Sanger sequencing. To construct a combined *speC* and *spd1* double exchange mutant the region upstream of *spd1* and the region downstream of *speC* were cloned either side of the pUCMUT *aphA3* cassette and introduced into Lineage C ST15 *emm*3 strain M3-C1. Mutagenesis was confirmed by PCR and Sanger sequencing. M3-C1 derivatives, M3-C1Δ*speC*, M3-C1Δ*spd1* and M3-C1Δ*speC/spd1*, were also subjected to whole genome sequencing (NCBI accession numbers SAMN05818659, SAMN05818660, SAMN05818661) which confirmed no other genetic changes had occurred compared with the M3-C1 parent strains except the targeted mutations.

### Assessment of iGAS patient outcome

Of the 442 whole genome sequenced *emm*3 collection isolated 2001–2013,^6^ 297 strains were from invasive disease cases. Laboratory records routinely obtained from patients in England with invasive GAS infection were submitted to the NHS Demographic Batch Tracing service to identify deaths, in accordance with approvals held by Public Health England. Results from the tracing were used to calculate 7 and 30 day case fatality rates based on the date culture positive specimens were taken. Data were then linked to strain sequence type and genome lineage. Lack of sufficient patient identifiers prevented the acquisition of mortality data for some cases.

### Statistical analysis

All statistical analyses were performed using GraphPad Prism software version 6.0 for windows; San Diego California USA. A *p*-value of ≤ 0.05 was considered significant.

## Results

### The ΦUK-M3.1 prophage increased the mitogenicity and DNase activity of Lineage C strains

**Figure 2.**
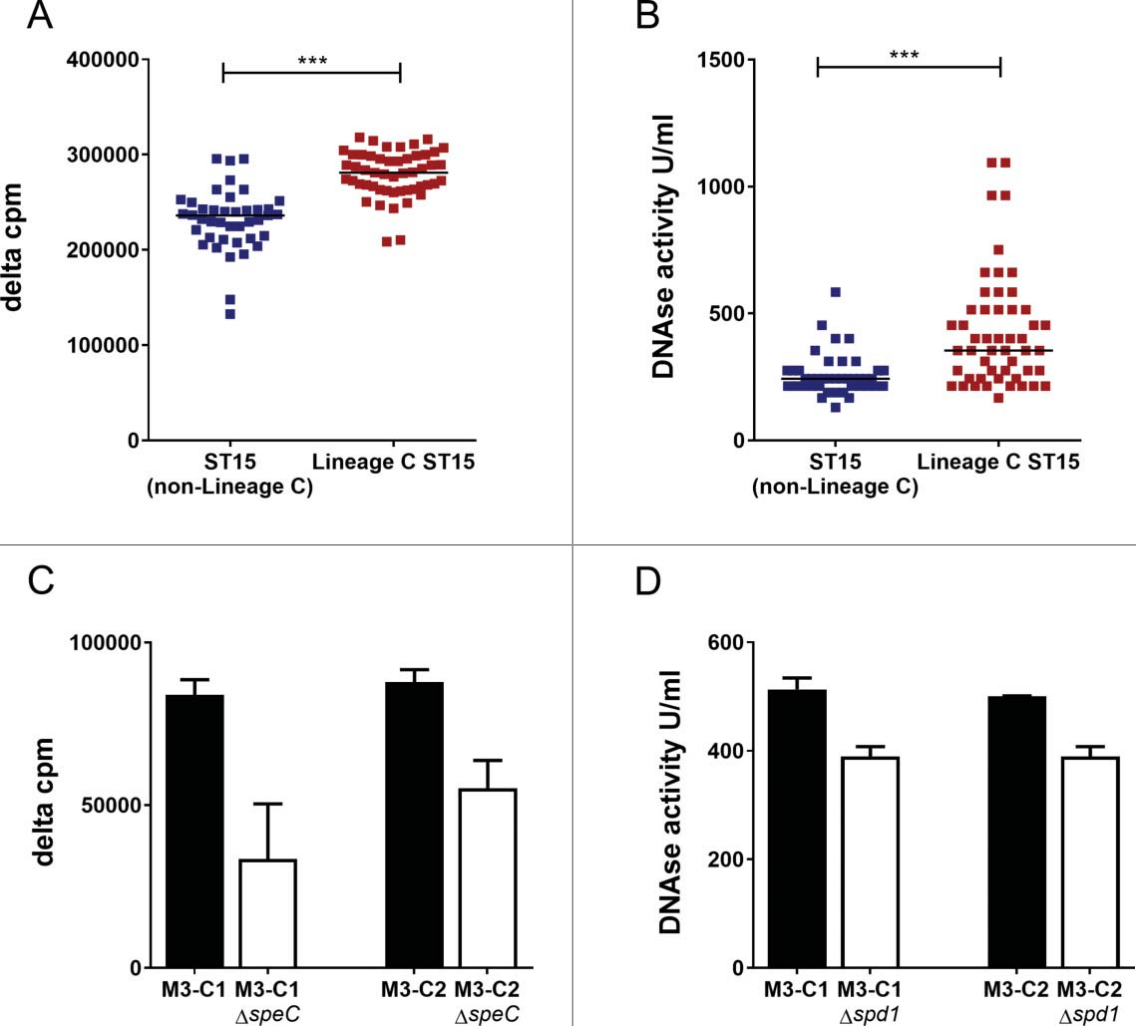
Lineage CST15 *emm*3 strains have greater DNase activity and enhanced mitogenicity compared with non-Lineage CST15 strains. (A) Human MNC proliferation was greater in response to broth culture supernatant of Lineage C ST15 strains (with ΦUK-M3.1-*speC*) (red, n = 51) compared with non-Lineage C ST15 strains (with Φ315.2-*ssa*) (blue, n = 41). Data represent counts per minute (cpm) of thymidine incorporation of a single human MNC donor minus the background level (delta cpm). All *emm*3 strains carried additional superantigens *speA, speG, speK* and the non-functional M3-*smeZ*. The experiment was repeated with a second donor and gave similar results. (B) DNase activity was greater in culture supernatant of Lineage C ST15 strains (with the ΦUK-M3.1-*spd1*) (red, n = 51) than non-Lineage C ST15 strains (blue, n = 41). Data represent DNase activity in units per ml of culture supernatant calculated relative to a standard. ***; *p* ≤ 0.0001 (Mann-Whitney). Horizontal line represents the median (C) Human MNC proliferation was reduced in response to culture supernatant of Lineage C ST15 strains M3-C1 and M3-C2 when *speC* was deleted by mutagenesis (M3-C1Δ*speC* and M3-C2Δ*speC*, white bars) compared with wild-type parental strains M3-C1 and M3-C2 (black bars). (D) DNase activity was reduced in culture supernatant of Lineage C ST15 strains M3-C1 and M3-C2 when *spd1* was deleted by mutagenesis (M3-C1Δ*spd1* and M3-C2Δ*spd1*, white bars) compared with wild-type parental strains M3-C1 and M3-C2 (black bars). Data represent the mean of 3 experimental repeats (+standard deviation).

The loss and acquisition of prophages had altered the superantigen and DNase complement of Lineage C ST15 strains compared with typical non-Lineage C ST15 strains. All Lineage C strains had lost the prophage Φ315.2 with the superantigen *ssa*, but the majority had also gained the prophage ΦUK-M3.1 which carries a superantigen, *speC* and a DNase, *spd1. In vitro* expression of SPEC by Lineage C strains was confirmed and measured by quantitative western blotting (median: 165 ng/ml, range: 81–377 ng/ml, n = 18 tested). Enhanced mitogenic activity toward human cells was detected in broth culture supernatant from Lineage C ST15 strains (all with ΦUK-M3.1-*speC*) compared with non-Lineage C ST15 strains (Fig. 2A), despite the presence of the superantigen gene *ssa* in the latter non-Lineage C strains. We identified no difference in SPEA production, measured by western blot, between Lineage C and non-Lineage C associated strains (Fig. S1)

*In vitro* DNase activity of Lineage C ST15 strains was also found to be greater compared with non-Lineage C ST15 strains (Fig. 2B). Furthermore, both mitogenic activity (Fig. 2C) and DNase activity (Fig. 2D) were reduced when deletions were made in *speC* or *spd1*, respectively, in 2 different Lineage C ST15 strains (M3-C1 and M3-C2) confirming that the activity was attributable to these genes.

### Evaluation of Lineage C strains during invasive infection in a superantigen-sensitive model

**Figure 3.**
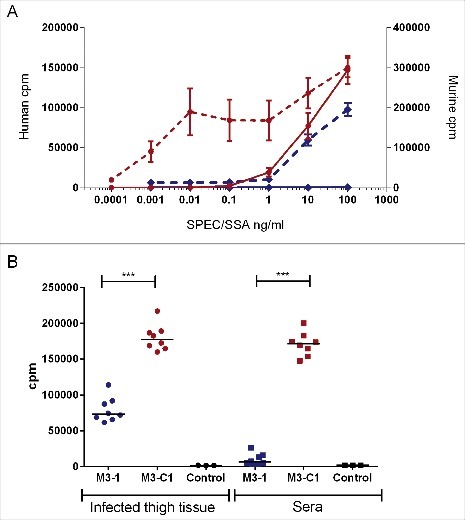
SPEC is more potent than SSA in both human and murine HLA-DQ8 transgenic cell culture and *in vivo*. (A) Human MNCs were stimulated with recombinant SPEC (red dotted line, left axis) or SSA (blue dotted line, left axis) at increasing concentrations and compared with murine splenocytes from HLA-DQ8 transgenic mice also stimulated with SPEC (red solid line right axis) or SSA (blue solid line, right axis). Data represent the mean ( ± standard deviation) of a single human donor measured in triplicate or splenocytes from 3 mice measured in triplicate. Human MNC data were reproduced in one other donor. (B) To see if this would be replicated *in vivo*, human MNCs were stimulated with infected thigh tissue (circles) or sera (squares) obtained from HLA-DQ8 mice after 24 h of infection with a non-Lineage C associated ST15 strain M3–1 (blue) or a Lineage C associated ST15 strain, M3-C1 (red); 8 mice per strain. A greater mitogenic response was observed when human MNCs were exposed to thigh or serum from mice infected with a Lineage C strain compared with a non-Lineage C strain. Control; uninfected mouse thigh tissue (black circles) or uninfected mouse serum (black squares). ***; *p* ≤ 0.0001 (Mann-Whitney). Each data point represents the mean of an individual mouse sample measured in triplicate. Horizontal lines represent the median.

Although the superantigen SPEC is preferentially presented to T cells by HLA-DR rather than HLA-DQ^12,13^ pilot studies showed that splenocytes from mice expressing HLA-DQ8 were more sensitive to SPEC than mice expressing HLA-DR4 (not shown). Spleen cells from HLA-DQ8 mice proliferated in response to SPEC in a dose-responsive manner, albeit at concentrations that were 3–4 orders of magnitude greater than concentrations required for human mononuclear cell (MNC) response (Fig. 3A). In contrast, both human cells and mouse cells responded poorly to SSA.

Having established that HLA-DQ8 transgenic mice can respond to SPEC, we infected HLA-DQ8 mice intramuscularly with *emm*3 ST15 strains that were representative of either Lineage C or the non-Lineage C. Tissue bacterial burden was not different between the 2 strains (Table S1), suggesting strains associated with Lineage C were not inherently more invasive than other ST15 strains. This was consistent with pilot experiments that screened multiple lineages of *emm*3 GAS in *Galleria mellonella*, an invertebrate model previously used effectively to screen for aggressive disease phenotypes.^14,15^ These preliminary studies showed wide variation in lethality but no particular association of Lineage C isolates with virulence; indeed, there was a trend for reduced lethality associated with Lineage C when compared with non-lineage C ST15 isolates (Fig. S3).

Mitogenic activity in the thigh tissue and sera of mice infected with a Lineage C strain (M3-C1) was significantly greater than non-Lineage C ST15 strain (M3–1) infected mice, confirming production of bioactive superantigen *in vivo* (Fig. 3B). Despite this, HLA-DQ8 mice did not generate a cytokine/chemokine response to SPEC that matched the observed difference in bioactive mitogen detected following infection (Tables S2 and S3). Both groups of mice produced a general cytokine/chemokine response to infection compared with uninfected mice.

### Enhanced nasopharyngeal shedding of Lineage C strains during nasopharyngeal infection

**Figure 4.**
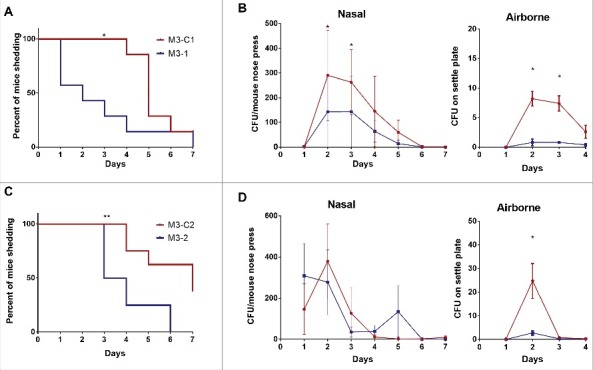
Nasopharyngeal infection with Lineage C strains resulted in prolonged nasal shedding and enhanced airborne shedding. Mice infected intranasally demonstrated direct nasal shedding of GAS over a longer period of time when infected with Lineage C strains (A) M3-C1 (red line) or (C) M3-C2 (red line) compared with mice infected with ST15 strains (A) M3–1 (blue line) or (C) M3–2 (blue line). Direct nasal shedding was monitored by daily nose press. *; *p* = 0.0167 (A) or **; *p* = 0.0062 (C) (Mantel-Cox Log-rank test). (B) Nasal shedding of GAS by each mouse was significantly greater in mice infected with M3-C1 (red line) compared with M3–1 (blue line) on day 2 (*p* = 0.0122) and day 3 (*p* = 0.021) of infection. Airborne shedding of GAS was also significantly greater in the cage of mice infected with M3-C1 compared with the cage of mice infected M3–1 on days 2 (*p* = 0.0294) and 3 (*p* = 0.0256). (D) Nasal shedding of GAS by each mouse was no different between those infected with M3-C2 compared with M3–2 but airborne shedding of GAS was significantly greater in the cage of mice infected with M3-C2 compared with the cage of mice infected M3–3 on day 2 (*p* = 0.0294). *; *p* ≤ 0.05 (Mann-Whitney). N = 7 per group. Nasal and airborne shedding data represent mean ( ± SEM).

Having established, experimentally, that Lineage C did not demonstrate increased invasiveness, experiments were conducted to evaluate fitness in nasopharyngeal infection again using HLA-DQ8 mice that are known to be sensitive to streptococcal superantigens.^16^ HLA-DQ8 mice were infected intranasally with either a Lineage C ST15 strain (M3-C1) or a non-Lineage C ST15 strain (M3–1). Nasal shedding of GAS was longitudinally monitored over 7 d by nose press as described previously.^11^ Mice infected with M3-C1 shed GAS for a longer period of time compared with mice infected with M3–1 (Fig. 4A) and nasally shed greater numbers of GAS on days 2 and 3 (Fig. 4B). Airborne shedding of GAS was also monitored in each cage and, from day 2, greater numbers were detected in the cage of M3-C1 infected mice compared with the cage of M3–1 infected mice (Fig. 4B). The prolonged nasal and greater airborne GAS shedding demonstrated by Lineage C M3-C1 compared with non-Lineage C M3–1 was maintained even when mice were infected with a 10-fold lower dose (∼10^6^ CFU) (Fig. S4).

Similar results were obtained using a different pair of M3 strains, M3–2 and M3-C2. Mice infected with Lineage C strain M3-C2 demonstrated prolonged nasal shedding compared with the non-Lineage C strain M3–2 (Fig. 4C). Although, in this instance, the number of nasal GAS shed by each mouse was not statistically significantly different between the 2 strains (Fig. 4D), greater numbers of airborne GAS shedding were detected when mice were infected with the Lineage C strain compared with the non-Lineage C strain (Fig. 4D).

**Figure 5.**
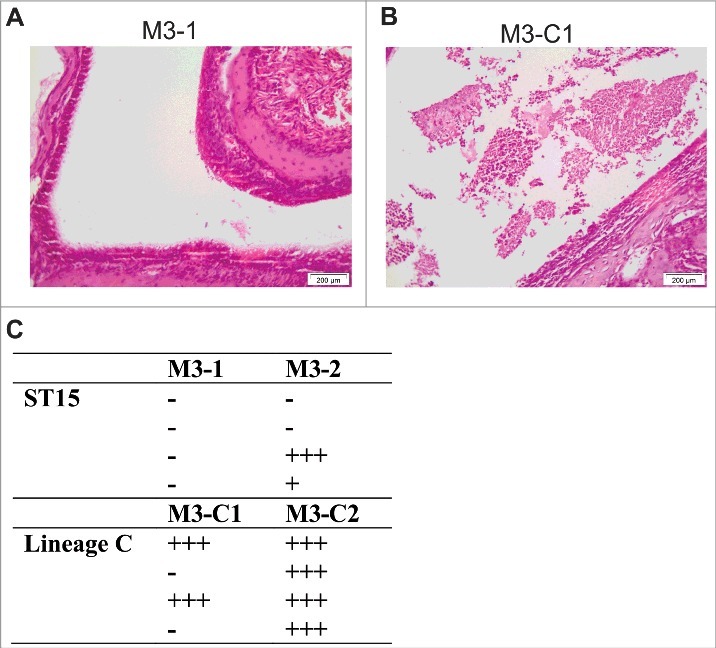
Nasopharyngeal infection with Lineage C associated ST15 strains resulted in enhanced nasal mucosal damage. Four mice were infected intranasally for 2 d with either one of 2 ST15 strains, M3–1 or M3–2, or one of 2 Lineage C-associated ST15 strains, M3-C1 or M3-C2. (A) and (B) photomicrographs of haemotoxylin and eosin stained nasal cavity sections following infection with either a non-Lineage C ST15 strain (A) or a Lineage C ST15 strain (B). Damage to the nasal mucosa with surface neutrophilic exudate was observed following Lineage C strain infection. (C) Semi quantitative histopathological analysis of all sections was performed to assess the damage to the nasal cavity based on level of rhinitis and associated neutrophilic exudate within the nasal cavity. +++; marked, ++; moderate, +; mild, −; no abnormality. Each row in each column represents an individual infected mouse. Free and phagocytosed bacteria were observed in all sections that scored above ‘no abnormality’.

To determine the mechanism that might underlie prolonged nasal shedding, sections of the nasopharynx were examined after 2 d of infection by a histopathologist (KS) and scored. Six of the 8 mice infected with non-Lineage C ST15 strains (M3–1 or M3–2) demonstrated no inflammation within the nasal cavity (Fig. 5A and C). In contrast, both Lineage C strains (M3-C1 or M3-C2) induced inflammatory changes in the nasal cavity (Fig. 5B) with moderate to marked rhinitis present in the majority of infected mice (6/8) (Fig. 5C).

### Enhanced nasal and airborne shedding of Lineage C strains is in part due to DNase *spd1*

**Figure 6.**
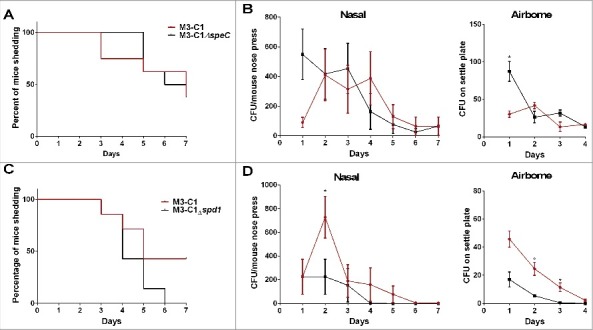
The DNase Spd1 contributes to nasal and airborne shedding of Lineage C strains. HLA-DQ8 transgenic mice were infected intranasally with either the parental wild-type strain (M3-C1, red line) or the *speC* deleted strain of M3-C1 (M3-C1Δ*speC* black line) and nasal shedding was monitored daily over a period of 7 d (A) along with the number of nasal GAS shed by each mouse and airborne GAS shed (B). N = 8 per group. HLA-DQ8 transgenic mice were also infected intranasally with either the parental wild-type strain (M3-C1, red line) or the *spd1* deleted strain of M3-C1 (M3-C1Δ*spd1*, black line) and nasal shedding was monitored daily over a period of 7 d (C) along with the number of nasal GAS shed by each mouse and airborne GAS shed (D). N = 7 per group. Deletion of *speC* had limited effect on the nasal infection with, in fact an increase in airborne GAS shedding of M3-C1Δ*speC* on day 1 (*p* = 0.0286). Deletion of the DNase *spd1* significantly reduced the number of nasal GAS shed by each mouse on day 2 and reduced airborne GAS shedding on days 2 (*p* = 0.0284) and 3 (*p* = 0.0275). *; *p* ≤ 0.05 (Mann-Whitney). Nasal and airborne shedding data represent mean ( ± SEM).

The prolonged nasal shedding and enhanced inflammation associated with Lineage C strains raised the possibility that the superantigen SPEC or the DNase may be in part responsible for this. Allelic replacement of *speC* or *spd1* was undertaken in the Lineage C strain M3-C1 to generate M3-C1Δ*speC* and M3-C1Δ*spd1*. Despite deletion of *speC*, nasal shedding of GAS (Fig. 6A) and number of nasal GAS shed (Fig. 6B) by infected mice remained unaffected. Airborne shedding of GAS likewise was unaffected by deletion of *speC* (Fig. 6B). In contrast, although deletion of the DNase *spd1* did not affect the length of time nasal shedding occurred (Fig. 6C), it reduced the number of nasal shed GAS on day 2 by ∼3-fold (Fig. 6D) and reduced airborne shedding by ∼5 and ∼10-fold on days 2 and 3 respectively (Fig. 6D). Deletion of both *speC* and *spd1* combined in M3-C1 also reduced airborne shedding (Fig. S5). To support these findings, deletion mutants of *speC* and *spd1* were also constructed in the second Lineage C strain M3-C2 yielding similar results consistent with an impact of *spd1* on airborne shedding (Fig. S6).

### Clinical severity phenotype associated with Lineage C strains

To determine if our *in vivo* findings related to clinical disease during the upsurge, we submitted all 297 *emm*3 invasive diseases cases (from the total of 442 sequenced isolates) (2001–2013) for patient outcome tracing. Patient status was available for 209 of the isolates, of which 58 were identified as having died within 7 d of diagnosis (28%) and 67 within 30 d (32%) (Table S4). Case fatality rates were similar in patients infected with *emm*3 ST15 strains compared with other *emm*3 STs both at 7 d (26% *v* 29%; χ^2^_(1 df)_ = 0.1790, *p* = 0.672) and 30 d (32% *v* 32%; χ^2^_(1 df)_ = 0.0183, *p* = 0.892) after diagnosis. Patients infected with Lineage C ST15 strains had slightly reduced survival than patients infected with non-Lineage C ST15 strains, although this did not reach statistical significance either at 7 d (28% *v* 24%; χ^2^_(1 df)_ = 0.1740, *p* = 0.677) or 30 d (35% *v* 29%; χ^2^_(1 df)_ = 0.4236, *p* = 0.515) after diagnosis.

### Discussion

The UK-wide upsurge in *emm*3 iGAS was largely accounted for by a new epidemic Lineage (Lineage C) of ST15 *emm*3, characterized by a different prophage profile associated with the loss of *ssa* and gain of *speC* and *spd1.*^6^ Despite a very high overall 30 day mortality observed due to *emm*3 GAS, in this study we have shown that Lineage C was not inherently more invasive or lethal. Instead, Lineage C strains were better able to cause prolonged nasopharyngeal infection and airborne shedding. Furthermore, we identified a potential role for the prophage-associated DNase Spd1 in airborne spread.

The presence of prophage ΦUK-M3.1 in Lineage C conferred increased mitogenicity, linked to SPEC production, and increased DNase activity *in vitro* and also *in vivo*. Streptococcal superantigens result in T cell activation through cross-linking of MHC class II and the T cell receptor (TCR) variable β subunit. Although mice are poorly responsive to superantigens, HLA class II transgenic mice can provide a superantigen-responsive model.^16^ To determine if SPEC might be responsible for increased disease due to Lineage C, we used HLA-DQ8 mice; contrary to published reports,^17^ spleen cells from mice expressing HLA-DQ8 could respond to SPEC, although the threshold for response was much greater than that demonstrated by human cells. As HLA-DR4 mice were unresponsive, the model represented the best available system to explore the potential role of SPEC during infection. Comparing non-isogenic, paired ST15 strains, we found no clear evidence that Lineage C was more invasive than non-Lineage C ST15 strains, consistent with the *Galleria mellonella* studies and clinical and epidemiological data. In contrast, we found that nasopharyngeal infection caused by Lineage C resulted in more prolonged nasal shedding, more abundant nasal shedding, and more airborne shedding of GAS than non-Lineage C strains.

Production of SPEA by *emm*18 GAS was recently linked to increased nasopharyngeal inflammation in HLA DQ8+DR4 transgenic mice^17^ and we therefore hypothesized that the phenotype observed was due to SPEC production. Unexpectedly, deletion of *speC* in 2 Lineage C-associated *emm*3 strains did not alter nasopharyngeal shedding or airborne shedding. The *emm*3 strains used in the current study also carry the superantigens *speA, speK, speG* and *smeZ*, however a conserved deletion in the *emm*3 *smeZ* results in a pseudogene that is non-functional as a mitogen.^8^ As HLA-DQ8 mice are highly sensitive to the streptococcal superantigens SPEA and SMEZ^16,18^ it is possible that any effect of SPEC was obscured by a much greater response to SPEA that is produced by both Lineage C and non-Lineage C strains in equal amounts. It seems likely that, in humans, the impact of SPEC on pharyngeal inflammation may be greater than we are able to demonstrate in the mouse model that has limited sensitivity to SPEC.

Surprisingly deletion of ΦUK-M3.1-associated *spd1* reduced airborne shedding, suggesting it may have played some role in the Lineage C nasopharyngeal infection phenotype. We predict that *spd1* expression is tightly associated with prophage induction, therefore accurate phenotype restoration may not be achieved with genetic complementation of the mutant. Reduction of airborne shedding was observed after nasopharyngeal infection with 2 different *spd1*-deleted Lineage C strains, and deletion of both *speC* and *spd1* confirmed this pattern. No other genetic changes were detected in the mutant strains compared with the parent strain. Others have shown that DNase is expressed during upper respiratory infection and may contribute to disease progression.^19,20^ The exact role for *spd1* in nasopharyngeal infection is unclear at this stage and is a subject of on-going work.

We considered the possibility that other genetic changes in Lineage C might account for the profound nasopharyngeal carriage phenotype. Prophage Φ315.1, which is absent in Lineage C strains compared with other ST15 strains, is not associated with any known virulence factors, but it lies within the only clustered regularly interspaced short palindromic repeat (CRISPR) region found in *emm*3 strains, and may render the CRISPR/cas system inactive.^21^ In addition to providing prokaryotic immunity to invading DNA, the CRISPR/cas system may also be involved in regulation of virulence gene expression,^22^ hence a possible impact of Φ315.1 presence or absence on virulence cannot be ruled out.

The sudden but transient nature of the upsurge suggested that introduction of the new prophage may have provided an advantage for Lineage C that could not be sustained, perhaps due to changes in population susceptibility. Lineage C may also not have been able to compete with other dominant non-*emm*3 lineages such as *emm*1 or the emergent variant of *emm*89 that were increasingly dominant in 2008–9.^1^

The emergence of Lineage C in the UK was not associated with increased mortality compared with other *emm*3 lineages, but was associated with a rise in invasive cases. There was a rise in scarlet fever notifications just before the 2008–9 upsurge^23^ and we speculate that increased non-invasive disease abundance in the community is the most likely basis for the observed rise in invasive disease; the experimental phenotype of Lineage C in the nasopharynx provides one explanation for a fitness advantage.

Notwithstanding the lack of any specific association of Lineage C with increased severity, invasive disease due to *emm*3 GAS overall had an extremely high attendant mortality of around 30% at 7 d in this study, exceeding that reported for *emm*1 iGAS.^24^ The reasons for the excessive mortality attributable to *emm*3 remain unknown, but underline the importance of better understanding the factors that govern *emm*3 disease abundance within the wider population.

## Supplementary Material

Supplementary Tables and Figures
