# Supplementary material for: Enhanced nasopharyngeal infection and shedding associated with an epidemic lineage of emm3 group A Streptococcus
Source: Virulence. 2017 May 1;8(7):1390–400. doi: 10.1080/21505594.2017.1325070 (PMC5711448; doi:10.1080/21505594.2017.1325070)
Supplement: Supplementary Tables and Figures [file kvir-08-07-1325070-s001.zip › KVIR_A_1325070_Supplement/Supplementary Figure 5[May9].docx]

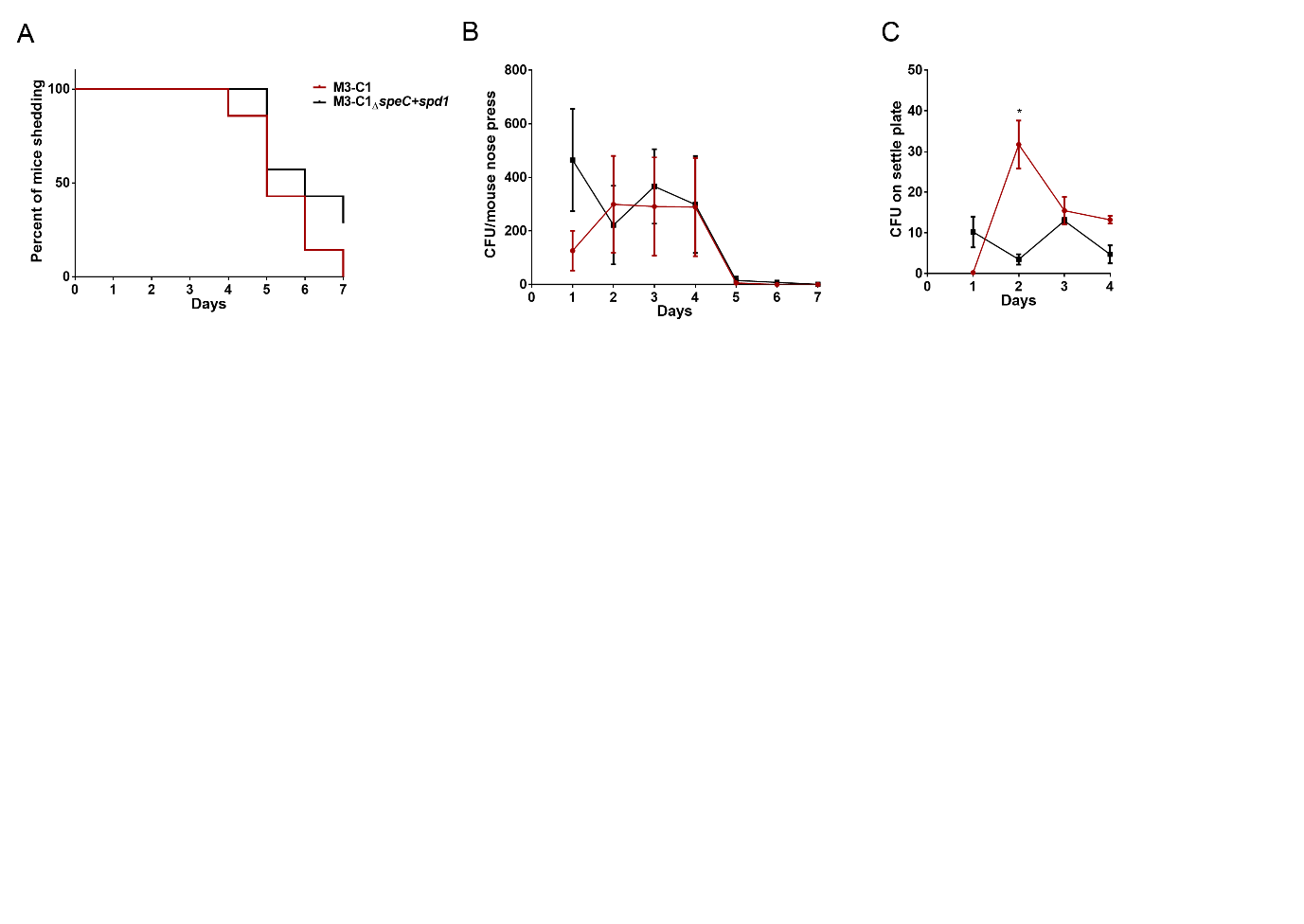


**Supplementary Figure 5. Deletion of *speC* and *spd1* combined reduced airborne shedding.** HLA-DQ8 transgenic mice were infected intranasally with either the parental wild-type strain (M3-C1, red line) or the *speC* and *spd1* deleted strain of M3-C1 (M3-C1Δ*speC+spd1*, black line) and nasal shedding was monitored daily over a period of seven days (**A**) as well as the number of nasal GAS shed by each mouse (**B**) and airborne GAS shed (**C**). Data represent the mean (±SEM) N=7 per group. Deletion of both *speC* and *spd1* significantly reduced airborne shedding on day 2 consistent with deletion of *spd1* alone. *; *p=*0.0284 (Mann-Whitney).
