# Supplementary material for: Enhanced nasopharyngeal infection and shedding associated with an epidemic lineage of emm3 group A Streptococcus
Source: Virulence. 2017 May 1;8(7):1390–400. doi: 10.1080/21505594.2017.1325070 (PMC5711448; doi:10.1080/21505594.2017.1325070)
Supplement: Supplementary Tables and Figures [file kvir-08-07-1325070-s001.zip › KVIR_A_1325070_Supplement/Supplementary Figure 6[May9].docx]

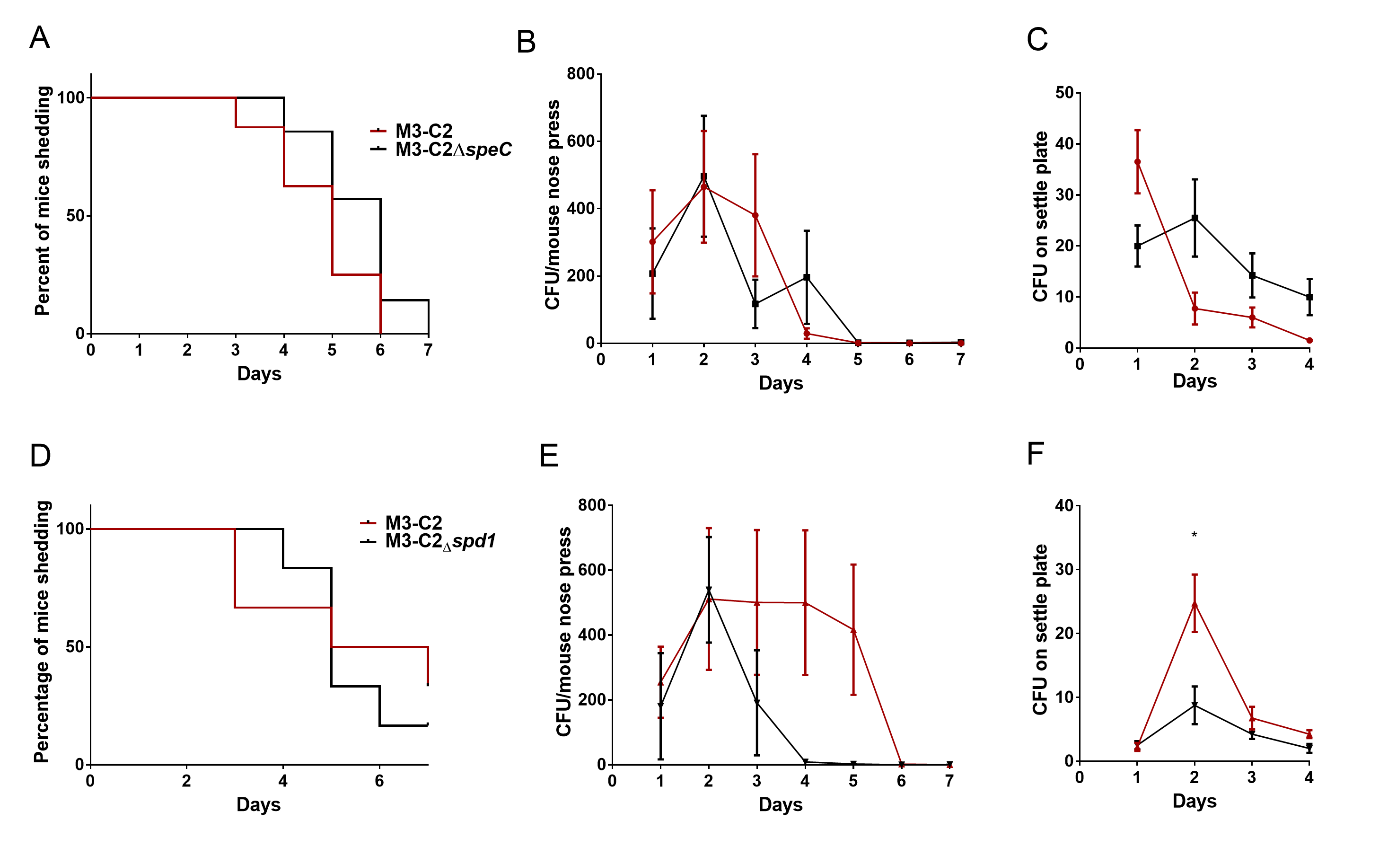


**Supplementary Figure 6. The DNase Spd1 contributes to nasal and airborne shedding of Lineage C strains.** HLA-DQ8 transgenic mice were infected intranasally with either the parental wild-type strain (M3-C2, red line) or the *speC* deleted strain of M3-C2 (M3-C2Δ*speC*, black line) and nasal shedding was monitored daily over a period of seven days (**A**) along with the number of nasal GAS shed by each mouse (**B**) and airborne GAS shed (**C**). N=7 per group. HLA-DQ8 transgenic mice were also infected intranasally with either the parental wild-type strain (M3-C2, red line) or the *spd1* deleted strain of M3-C2 (M3-C2Δ*spd1*, black line) and nasal shedding was monitored daily over a period of seven days (**D**) along with the number of nasal GAS shed by each mouse (**E**) and airborne GAS shed (**F**). N=7 per group. Deletion of *speC* had limited effect on the nasal infection. Deletion of the DNase *spd1* significantly reduced airborne shedding on day 2. *; *p* =0.0286 (Mann-Whitney).
