# Supplementary material for: Enhanced nasopharyngeal infection and shedding associated with an epidemic lineage of emm3 group A Streptococcus
Source: Virulence. 2017 May 1;8(7):1390–400. doi: 10.1080/21505594.2017.1325070 (PMC5711448; doi:10.1080/21505594.2017.1325070)
Supplement: Supplementary Tables and Figures [file kvir-08-07-1325070-s001.zip › KVIR_A_1325070_Supplement/Supplementary Table 2 [May9].docx]

**Supplementary Table 2. Levels of cytokines/chemokines in thigh muscle of infected HLA-DQ8 transgenic mice**

| **Factor** | **M3-1**  Median (range) pg/ml |  | **M3-C1**  Median (range) pg/ml | **Uninfected^a^**  Median (range) pg/ml |
| --- | --- | --- | --- | --- |
| IL-1α | 2891 (607.6-6896) |  | 1440 (1007-3317) | 6.4 (4.1-33.1) |
| IL-1β | 4942 (1591-12213) |  | 5280 (2273-8805) | UD^b^ |
| IL-2 | 13 (11.89-15.5) |  | 12.2 (10.9-15.7) | 12.7 (8.5-17) |
| IL-4 | 54.6 (27.5-75.2) |  | 53.0 (26.4-79.7) | UD |
| IL-5 | 86.1 (42.9-157.3) |  | 74.98 (43.7-138.7) | UD |
| IL-6 | 1686 (1287-2604) |  | 1912 (1265-2596) | 129.3 (87.9-239.8) |
| IL-10 | 285.5 (159.1-382.3) |  | 202.1 (149.3-330.6) | UD (UD-31.2) |
| IL-12 | 8.3 (UD-32.2) |  | 8.9 (UD-19.2) | UD |
| IL-13 | UD (UD-10. 7) |  | UD | UD |
| IL-17 | 11.7 (UD-23.4) |  | 5.88 (2.3-18) | UD |
| MCP-1 (CCL2) | 150.6 (79.3-366.4) |  | 136.4 (78.5-222.7) | UD |
| MIP1-α (CCL3) | 414 (109.3-634.1) |  | 320.4 (233.4-461.1) | UD |
| MIG (CXCL9) | 386.7 (109.2-1582) |  | 387.8 (126.6-1329) | UD |
| IP-10 (CXCL10) | 19.1 (5.9-130.5) |  | 20.4 (8.1-49.9) | UD |
| IFN-γ | 4.035 (UD-180.3) |  | UD (UD-36.1) | UD |
| TNF-α | 36.9 (11.8-53.9) |  | 31.48 (22.5-51.7) | UD |
| VEGF | 132.3 (34.3-303.8) |  | 98.2 (76.8-165.5) | 5.1 (0.4-8.7) |
| GM-CSF | 36.4 (18-64.7) |  | 29.9 (19.4-49.9) | UD |
| FGF | 4147 (2632-7298) |  | 4152 (3052-5334) | 4344 (3668-4369) |
| KC | 1194 (745.3-1814) |  | 1147 (880.8-1680) | UD |

^a^Thigh tissue obtained from uninfected HLA-DQ8 mice

^b^UD; Undetectable levels

No statistical difference was observed between the two strains for any cytokine/chemokine (two-tailed Mann-Whitney *p* > 0.05).
