# Supplementary material for: Enhanced nasopharyngeal infection and shedding associated with an epidemic lineage of emm3 group A Streptococcus
Source: Virulence. 2017 May 1;8(7):1390–400. doi: 10.1080/21505594.2017.1325070 (PMC5711448; doi:10.1080/21505594.2017.1325070)
Supplement: Supplementary Tables and Figures [file kvir-08-07-1325070-s001.zip › KVIR_A_1325070_Supplement/Supplementary Table 3[May9].docx]

**Supplementary Table 3. Levels of cytokines/chemokines in serum of intramuscularly infected HLA-DQ8 transgenic mice**

| **Factor** | **M3-1**  Median (range) pg/ml | **M3-C1**  Median (range) pg/ml | **Uninfected^a^**  Median (range) pg/ml |
| --- | --- | --- | --- |
| IL-1α | UD^b^ (UD-66.1) | UD (UD-75.8) | UD |
| IL-1β | 12.9 (UD-63.3) | 17.7 (UD-31.4) | UD |
| IL-2 | 26.2 (9.1-41.5) | 23.8 (20.2-41.2) | 10 (1.3-14.5) |
| IL-4 | 119.9 (79-589.4) | 155.5 (116.0-303.9) | 79 (59.8-92.4) |
| IL-5 | 75.5 (42.1-399.6) | 107.9 (39.9-344.7) | UD |
| IL-6 | 172 (110.5-225.5) | 175 (127.4-237.8) | UD |
| IL-10 | UD (UD-197.3) | UD | UD |
| IL-12 | 65.8 (19.4-129.7) | 79.9 (69.4-112.7) | 89.4 (82.8-89.8) |
| IL-13 | 6.4 (UD-345.3) | UD (UD-84.7) | UD |
| IL-17 | 4.3 (2.1-7.4) | 3.5 (2.2-6.3) | UD (UD-1.7) |
| MCP-1 (CCL2) | 125 (62.8-473.9) | 174.9 (77.6-577) | UD |
| MIP1-α (CCL3) | 20.4 (13.4-120.4) | 27.4 (19.1-74.5) | 13.4 (7.6-38.3) |
| MIG (CXCL9) | 2172 (248.2-3438) | 1576 (218.4-2406) | UD |
| IP-10 (CXCL10) | 522.1 (318.4-1573) | 722.3 (217.1-1185) | UD |
| IFN-γ | 114.7 (54.37-314.7) | 100.6 (8.9-210.2) | UD (UD-54.4) |
| TNF-α | UD | UD (UD-14.6) | UD |
| VEGF | 1.1 (UD-74.4) | 3.6 (UD-47.2) | UD |
| GM-CSF | 7.9 (UD-42.8) | 4.7 (UD-44.9) | UD (UD-12.7) |
| FGF | 978.9 (629-1358) | 1026 (931.5-1625) | 988.3 (420.1-1009) |
| KC | 2658 (348.6-7084) | 3935 (3314-6849) | UD |

^a^Sera obtained from uninfected HLA-DQ8 mice

^b^UD; Undetectable levels

No statistical difference was observed between the two strains for any cytokine/chemokine (two-tailed Mann-Whitney *p* > 0.05).
